# Mental Health Nurse’s Exposure to Workplace Violence Leads to Job Stress, Which Leads to Reduced Professional Quality of Life

**DOI:** 10.3389/fpsyt.2018.00059

**Published:** 2018-02-27

**Authors:** Michal Itzhaki, Irit Bluvstein, Anat Peles Bortz, Hava Kostistky, Dor Bar Noy, Vivian Filshtinsky, Miriam Theilla

**Affiliations:** ^1^Nursing Department, School of Health Professions, Sackler School of Medicine, Tel-Aviv University, Tel Aviv, Israel; ^2^The Herczeg Institute on Aging, Tel Aviv University, Tel Aviv, Israel; ^3^Nursing Management, Sheba Medical Center, Ramat Gan, Israel; ^4^Sha’ar Menashe Mental Health Center, Emeq Hefer, Israel

**Keywords:** workplace violence, professional quality of life, job stress, mental health nurses, compassion satisfaction, compassion fatigue

## Abstract

Professional quality of life (ProQOL) reflects how individuals feel about their work as helpers. Psychiatric ward nurses cope with significant psychological and physical challenges, including exposure to verbal and physical violence. This study was based on two aspects of ProQOL, the positive compassion satisfaction, and the negative compassion fatigue, with the aim of investigating the relation of ProQOL to job stress and violence exposure at a large mental health center. Data were collected from 114 mental health nurses (49/63 M/F) who completed a self-administered questionnaire examining violence exposure, ProQOL, and job stress. The results showed that during the last year, almost all nurses (88.6%) experienced verbal violence, and more than half (56.1%) experienced physical violence. Only 2.6% experienced no violence. ProQOL was not associated with violence exposure but was reduced by work stress and by previous exposure to violence; nurses who perceived their work as more stressful had lower satisfaction from their work. In conclusion, although most mental health nurses are exposed to physical and verbal violence, their ProQOL is more related to job stress than to workplace violence (WPV). Hospital managements should conduct work stress reduction intervention programs and promote strategizes to reduce WPV. Further exploration of (a) factors affecting ProQOL and (b) the effect of violence coping workshops on ProQOL is warranted.

## Introduction

Workplace violence (WPV) toward nurses working in the hospital environment is a well-known issue worldwide (1, 2). In fact, in a literature review conducted by Nowrouzi and Huynh (3) on the 50 most cited WPV-related articles, it was found that 46.4% of the sampling population in these top articles involved nurses.

Anderson (4) stated that nurses in psychiatric wards and emergency areas are at the highest risk of suffering from WPV, probably because the necessarily high level of nurses–patient contact increases the exposure of the nurse to the hazard. By contrast, Whittington and Wykes (5) proposed a cyclical model of violence to mental health nurses, and suggested that nurses who were relatively unavailable to the patients were at a higher risk of assault, whilst nurses who spent more time in the patients’ area were at lower risk. According to this model, experiencing an assault will cause the victim, in our case the nurse, to develop a post-traumatic stress response, which will influence the nurse’s mental health. Moreover, according to Whittington and Wykes (5) there are two main aspects of nurse behavior that may generate patient anger and continue the circular model: social distance between the nurse and the patient, and behavior that the patients find annoying. Another explanation for the high rate of WPV against mental health nurses suggests that nurses’ perception of poor organizational justice and poor teamwork can negatively affect staff-patient interaction, which then promotes increased violent assaults by patients (6).

The prevalence of WPC in nursing undoubtedly influences job performance, recruitment, desire to stay in nursing (7, 8) and the overall professional quality of life (ProQOL) (9–11). ProQOL, a model suggested by Stamm (12), consists of two aspects (positive and negative) that affect the life of professional caregivers. The positive aspect includes compassion satisfaction (CS) which reflects the positive feelings gained from helping others. Compassion fatigue (CF), the negative aspect is further divided into (a) burnout (BO), which reflects the emotional exhaustion, frustration, feelings of hopelessness and difficulties dealing with the job, and (b) secondary traumatic stress (STS) which is the result of work-related secondary exposure to people who have suffered from a traumatic event.

WPV toward nurses has been shown to cause both BO and STS (13, 14). A Korean study examining the effect of WPV against nurses, on ProQOL and staff turnover, found that the highest rates of all three types of violence (verbal abuse, physical threats, and physical violence), were against nurses in the psychiatric unit. Nurses who experienced all three types of violence had the highest rate of secondary trauma level of ProQOL. Staff turnover was increased for nurses who suffered verbal abuse or physical threats (9).

WPV is also a major contributor to work stress among nurses (8, 15, 16), which has a negative impact on job performance, job satisfaction (16–18) and leads to BO (19). Nurses working in a psychiatric setting have a higher level of work stress compared to nurses working in a general hospital (20) and are therefore at higher risk for the symptoms derived from work stress.

Life satisfaction is considered to be a good indicator for subjective well-being (21, 22), and is certainly associated to work life satisfaction (23).

The present study was designed to focus on the life satisfaction of mental health nurses with respect to work-related aspects: ProQOL, job stress, and WPC, and to investigate the effect of job stress, and exposure to violence on nurses’ ProQOL. Based on the literature, we hypothesized that exposure to verbal and/or physical violence would be a major contributor of work stress and would be related to low CS, and high CF.

## Materials and Methods

### Sample

A descriptive, cross-sectional correlative study was conducted to explore the association between violence exposure, job stress, and ProQOL. A convenience sample of nurses was recruited from a large mental health center in Israel, which contains 520 beds and numbers 230 mental health nurses. The nurses worked in various open and closed departments: emergency, rehabilitation, psychogeriatric, long-term care (including three closed departments), acute illnesses (including three closed and one open departments), and a national forensic security ward (including four closed departments).

### Tools

Each participant completed a self-report structured questionnaire. Written informed consent was obtained from all participants. The questionnaire included demographic parameters, such as age, sex, state of birth, and marital status. In addition, the questionnaire also included professional details; working department, length of employment as a nurse (years), years of employment in the department, professional education, participation in a violence workshop at the workplace, and full-time equivalent.

### Measures

#### Violence Exposure

Violence exposure was measured by the method developed previously by Itzhaki et al. (24). The variables were measured by four questions, namely during your work, have you been exposed to physical violence from a patient and/or his/her family in the last year? During your work, have you been exposed to verbal violence from a patient and/or his/her family in the last year? Throughout your years of nursing practice, have you ever been exposed to physical violence from a patient and/or his/her family? Throughout your years of nursing practice, have you ever been exposed to verbal violence from a patient and/or his/her family? Respondents were asked to rate their answer on a 5-point Likert scale from 1 (not at all) to 5 (very often). The final score for violence exposure was calculated as the average of the four questions. The Alpha Cronbach value of Itzhaki et al. (24) was 0.88 and was 0.87 in the present study.

#### ProQOL Scale

Professional quality of life scale measurement of the quality of life of the nurse was based on a scale developed by Stamm (12), which comprises 30 items assessing CS, and two measures of CF: BO and secondary stress. The questionnaire was translated according to Brislin (25) in the following manner: (a) the questions were translated into Hebrew, (b) retranslated into English by a native English speaker, (c) the back translation was then compared to the original to confirm the Hebrew translation. The Cronbach’s alpha value for the original ProQOL questionnaire was 0.88 (12).

Due to a low reliability score, questions 4, 15, and 29, which all measure BO, where removed. This resulted in reliability scores of 0.90, 0.62, and 0.85 for CS, BO, and STS, respectively, in the Hebrew version.

#### Job Stress in the Last Month

Job Stress in the last month: the nurses’ job stress was measured by a question developed by Shen et al. (26), asking the respondents to rate their job stress in the last month on a scale from 1 (not at all) to 5 (very often). The question was translated by Itzhaki et al. (24). It has been shown in previous studies that a single item can measure stress reliably (26–28).

### Ethical Considerations

The study was approved by the mental health center ethics committee.

### Data Analysis

Descriptive statistics were generated for all variables. Pearson correlation coefficients were performed in order to examine the relationships between study variables, and Students *t*-tests were used to examine differences between dichotomous groups (i.e., gender, department type, participation in a violence workshop). Multiple linear regression analyses were conducted using the enter method to examine how much of the nurse’s ProQOL could be explained by independent variables. Statistical significance was set at 0.05 and the data were analyzed using IBM SPSS software version 24 (SPSS Inc., Chicago, IL, USA).

## Results

The sociodemographic information is presented in Table 1. A total of 114 nurses were recruited (response rate = 50%). The age of the nurses ranged between 26 and 64 years old (M = 47.3; SD = 9.02) with 49 males (43.8%), and 63 females (56.4%). The majority of the sample nurses were born in Israel or Russia (*n* = 102, 89.5%) and were married (*n* = 88, 77.2%). Most of the nurses (*n* = 100, 87.7%) worked full-time and had been employed as nurses for at least 10 years (*n* = 87, 76.3%). With regard to nursing qualifications, 18.4% (*n* = 21) of the respondents were practical nurses, while 34.2% (*n* = 39) had a Diploma in Nursing, 36% (*n* = 41) had a Bachelor of Nursing, and 6.2% (*n* = 7) held a Master or PhD Degree. About half the nurses worked in closed departments (*n* = 55, 48.2%). Almost all the nurses (88.6%) had experienced verbal violence in the last year and more than half (56.1%) had experienced physical violence in the last year. Overall, 97.6% of the nurses had experienced at least one type of violence during their career, and most (*n* = 78, 68.4%) had participated in a workshop for coping with violence.

**Table 1:** Sociodemographic characteristics of the participants (*n* = 114).

Variable	Overall
Age in years M (SD)	47.3 (±9)
Gender	*N* (%)
Female	63 (56.3)
Male	49 (43.8)
Missing	2 (1.8)
**Country of birth**	
Israel	61 (53.5)
Russia	41 (36)
Other	5 (4.4)
Missing	7 (6.1)
**Marital status**	
Married	88 (77.2)
Divorced	16 (14)
Single	4 (3.5)
Widowed	1 (0.9)
Missing	5 (4.4)
**Full/part-time**	
Full-time	100 (87.7)
Part-time	9 (7.9)
Missing	5 (4.4)
**Wards**	
Acute illnesses	11 (9.6)
Forensic	37 (32.4)
Psychogeriatric	11 (9.6)
Emergency	4 (3.5)
Missing	16 (14)
**Years of employment as a nurse**	
1–5	7 (6.1)
6–10	5 (4.4)
>10Missing	87 (76.3)15 (13.1)
**Years of employment in the department**	
<1	1 (0.9)
1–5	44 (38.6)
6–10	25 (21.9)
>10	26 (22.8)
Missing	18 (15.7)
**Participation in violence workshop**	
Yes	78 (68.4)
No	23 (20.2)
Missing	13 (11.4)

*RN, Registered Nurse; BA, Bachelor of Art; PhD, Doctor of Philosophy*.

*T*-tests revealed that exposure to physical and verbal violence was more frequent in closed wards than in the open ones (M_(closed)_ = 2.3, SD_(closed_) = 1.2*;* M_(open)_ = 1.74, SD_(open)_ = 0.97, *t*_(107)_ = 2.61, *p* < 0.05 for physical violence; M_(closed)_ = 3.3, SD_(closed_) = 1.1*;* M_(open)_ = 2.7, SD_(open)_ = 1.2, *t*_(108)_ = 2.48, *p* < 0.05 for verbal violence). There were no significant associations between violence exposure, work stress or ProQOL with the nurses’ gender or participation in a violence workshop.

**Figure 1:**
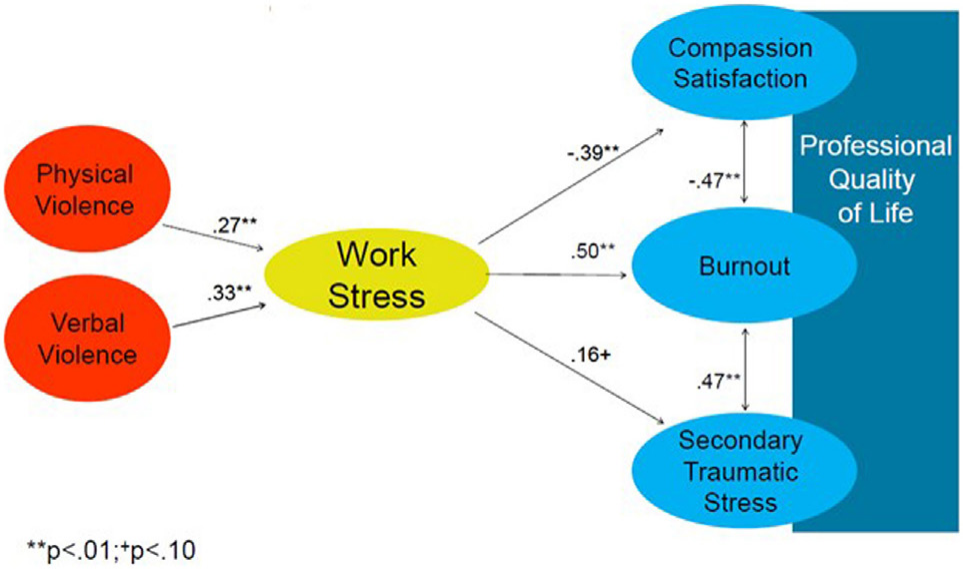
Main relationships of the study objectives: violence is associated with work stress, while work stress is associated with professional quality of life (proQOL). No direct or mediated association between violence exposure and proQOL was found.

A correlational analysis of the research variables is presented in Table 2. Within the three measures of ProQOL, burnout was negatively correlated with CS (*r* = −0.47, *p* < 0.01), and positively correlated with STS (*r* = 0.47, *p* < 0.01), while no association was found between CS and STS. With regard to violence, exposure to physical violence was positively and highly correlated with exposure to verbal violence (*r* = 0.65, *p* < 0.01). Interestingly, age and seniority as a nurse were negatively associated with exposure to physical violence (*r* = −0.31, *p* < 0.01, *r* = −0.35, *p* < 0.01 respectively) and verbal violence (*r* = −0.29, *p* < 0.01, *r* = −0.34, *p* < 0.01, respectively). Nurses who perceived their work as more stressful also experienced higher levels of burnout (*r* = 0.501, *p* < 0.01) and lower levels of CS (*r* = −0.39, *p* < 0.01). That is, the higher the work stress, the lower the nurse’s satisfaction from helping others. Unexpectedly, work stress was not associated with STS. While the two types of violence were positively correlated with job stress (verbal violence: *r* = 0.27, *p* < 0.01; physical violence: *r* = 0.33, *p* < 0.01), exposure to neither physical nor verbal violence was associated with CS and CF (i.e., burnout and STS). Nonetheless, exposure to either physical or verbal violence was positively correlated with work stress (*r* = 0.27, *p* < 0.01, *r* = 0.33, *p* < 0.01 respectively). The main significant relationships are described in Figure 1.

**Table 2:** Correlations between violence exposure, job stress, ProQOL, and age and seniority (*n* = 114).

Variable	1	2	3	4	5	6	7
1. Physical violence	–						
2. Verbal violence	0.65**	–					
3. Work stress	0.27**	0.33**	–				
4. Compassion satisfaction (ProQol)	0.02	−0.13	−0.39**	–			
5. Burnout (ProQol)	0.08	0.16	0.50**	−0.47**	–		
6. Secondary traumatic stress (ProQol)	0.05	0.02	0.16	−0.10	0.47**	–	
7. Age	−0.31**	−0.29**	−0.22*	0.08	−0.16	−0.09	–
8. Years as nurse	−0.35**	−0.34**	−0.18	0.07	−0.18	−0.14	0.85**

*ProQol, professional quality of life*.

*p < 0.05.

**p < 0.01.

In order to examine the unique contribution of physical violence, verbal violence, job stress, job seniority, and type of ward, to the ProQOL, we conducted multiple linear regression analyses. The model, including all variables, could explain 21% of the variance in CS, and 26% of the variance in BO. Interestingly, the only parameter found to be associated with these variables was work stress (for CS β = −0.47, *p* < 0.01; for BO β = −0.59, *p* < 0.01). Exposure to violence, job seniority, and type of ward were not significantly related to either CS or BO. The model for STS was not significant.

## Discussion

The purpose of this study was to examine the effects of WPV toward nurses working in psychiatric wards on work stress, CS, and CF. As hypothesized, the results show that exposure to verbal and/or physical violence is associated with work stress. Jackson et al. (8) suggested that exposure to violence causes feelings of unsafety, which enhances other work stressors. However, Hauge et al. (29) found that stress in the workplace environment may arouse aggression, hence in this case, work stress is the cause rather than the result. This may create a cycle of violence and work stress, that agrees with Whittington and Wykes (5)’s cyclical model of violence mentioned above, and is supported by the findings that work environment has a major role in the rate of violent incidents (30). According to Itzhaki et al. (24), the unsafe work environment of mental health nurses not only impacts work stress but also reduces life satisfaction. This last effect might be counterbalanced by enhancing feelings of security and support, as well as promoting collaboration between the nursing team (31).

Interestingly, the results of the current study indicated that exposure to violence had no effect on the ProQOL of mental health nurses. This result is in accordance with the findings reported by Itzhaki et al. (24) that life satisfaction is not affected by WPC but rather by work stress. This surprising result may be explained by social tolerance of violence toward nurses, many of whom, especially mental health nurses, believe that violence is an integral component of their job (24, 32–34). In other words, the absence of association between violence exposure to ProQOL may be viewed as an adjusted reaction to the specific work characteristics in a mental health center.

Another possible explanation is that our sample population exercised evidence-based practices. According to Craig and Sprang (35), the application of evidence-based practices establishes conditions where nurses feel more equipped to treat patients, thereby decreasing CF and increasing CS. In the current study, the effect of exposure to violence may have been mitigated by the hospital treatment strategy. The majority of the nurses (68.4%) in the sample stated that they had participated in an annual 1-day program workshop for violence coping, conducted by the mental health hospital. Similar training programs aimed at developing interpersonal skills and behavior management practices to intervene in violence conditions have been shown to have a positive effect on mental health workers’ psychological distress, feelings of safety, and coping confidence (36).

Finally, on a more technical level, since most of the nurses experienced violence at the workplace, there may not be enough variance in the violence exposure variable to reveal the statistical associations with other measures.

Another finding raised by the current study was the high positive correlation between exposure to physical and verbal violence. One explanation is that the exposure to either verbal or physical violence may generate poor interpersonal relationships, a factor that has been shown, by Camerino et al. (15), to give rise to more violence. A study, by Anderson (4), also supported these results, although in this case the violence was associated with the behavior of the victim, and specifically, to the victim’s perception of the violent situation. The negative correlation between age and seniority and exposure to physical and verbal violence, presented in our study results, is also supported by previous studies (37–39), which attributed this factor to their vulnerability. Moreover, in the current study, violence exposure was higher in closed wards, a finding that has been attributed to the danger, uncertainty, and stress resulting from the frequency of aggression toward staff commonly reported by mental health workers working in closed wards (31). The present study also showed that ProQOL is affected by work stress. Examining the relationship between work stress and BO, Wu et al. (40) suggested that work-related stress can be reduced by intervention programs that enhance nurse’s coping resources and by doing so, decrease BO. When looking at the cost of such intervention programs, it is important to consider the high loss of hours attributed to violence (8, 41). According to Hoel et al. (42), it is cost saving for organizations to hold such intervention programs when considering the losses attributed to stress and violence.

The low reliability score of the proQOL scale observed, and the decision to remove items 4, 15, and 29 from the BO section, are supported by the study of Hemsworth et al. (43) who showed that the same items had low reliability scores when a proQOL questionnaire was compared across three different populations. This consistency raises the need to investigate improvements that could be introduced into the proQOL instrument, The main limitation of this study is the size and sample population, obtained from a single mental hospital in Israel, which may not be representative of all other mental health hospitals. In addition, the response rate was moderate (50%). This may be due to the low response rate yield in voluntary questionnaire, spatially when the sample group is nurses who are preoccupied with their work. The nurses who agreed to participate and signed the informed consent form were given questionnaires to complete. The time taken to complete the questionnaire was 15 min.

Some of the nurses who did not fill out the questionnaires claimed that they did not have time to fill the questionnaire, which was distributed during the work. Some of the nurses also noted that they did not wish to reveal personal aspects, such as their exposure to violence.

The heterogeneity within the study sample may also be a limitation that makes it more difficult to determine the influence on other factors in the response. The information of caseload was not obtained from participants, this information may have influence on the results. Lastly, the study collected the data retrospectively (the questions referred to the last 12 months). The results depend upon the ability of the participants to recall events.

## Conclusion

Despite the high prevalence of physical and/or verbal violence directed toward them, the ProQOL of mental health nurses is more affected by work stress than by WPC. However, exposure to violence increase work stress and, thus, there is an indirect relationship between work place violence and proQOL through work stress. Since work stress is correlated to low efficacy, absenteeism, emotional burden, and illness, hospital managements should conduct stress reduction intervention programs focusing on the development of self-awareness of stress implications, and increasing cognitive self-control (44). Moreover, policymakers and employers should lead strategies to reduce WPC by giving more prioritization to staff safety, and developing procedures to protect health care workers through safety regulators and enforcement mechanisms (45).

We recommend that after a violent incident, the nurse’s attitudes toward the violent patient should be examined in order to examine whether the reason that exposure to WPC does not influence nurse’s ProQOL is due to an adjusted reaction to characteristics of work in a mental health center.

## Ethics Statement

The study was approved by Sha’ar Menashe Mental Health Center ethics committee, and participating nurses signed a consent form. The consent forms and the completed questionnaire were separated, and data were treated confidentially.

## Author Contributions

All authors meet the criteria for authorship. MI, IB, and AP contributed to the conception and design of the study, acquisition of data, analysis and interpretation of data, revising it, and final approval of the version to be submitted. MT contributed to the conception and design of the study, drafting the article, revising it, and final approval of the version to be submitted. HK, DN, and VF acquisition of data, analysis, and interpretation of data.

## Conflict of Interest Statement

The authors declare that the research was conducted in the absence of any commercial or financial relationships that could be construed as a potential conflict of interest.
